# Substrate Selection for Fundamental Studies of Electrocatalysts and Photoelectrodes: Inert Potential Windows in Acidic, Neutral, and Basic Electrolyte

**DOI:** 10.1371/journal.pone.0107942

**Published:** 2014-10-30

**Authors:** Jesse D. Benck, Blaise A. Pinaud, Yelena Gorlin, Thomas F. Jaramillo

**Affiliations:** Department of Chemical Engineering, Stanford University, Stanford, California, United States of America; RMIT University, Australia

## Abstract

The selection of an appropriate substrate is an important initial step for many studies of electrochemically active materials. In order to help researchers with the substrate selection process, we employ a consistent experimental methodology to evaluate the electrochemical reactivity and stability of seven potential substrate materials for electrocatalyst and photoelectrode evaluation. Using cyclic voltammetry with a progressively increased scan range, we characterize three transparent conducting oxides (indium tin oxide, fluorine-doped tin oxide, and aluminum-doped zinc oxide) and four opaque conductors (gold, stainless steel 304, glassy carbon, and highly oriented pyrolytic graphite) in three different electrolytes (sulfuric acid, sodium acetate, and sodium hydroxide). We determine the inert potential window for each substrate/electrolyte combination and make recommendations about which materials may be most suitable for application under different experimental conditions. Furthermore, the testing methodology provides a framework for other researchers to evaluate and report the baseline activity of other substrates of interest to the broader community.

## Introduction

The selection of an appropriate substrate is an important preliminary step in accurately evaluating electrochemically active materials including electrocatalysts, photoelectrodes, and electrochemical capacitors [1]. The substrate is typically defined as an inert, electrically conductive support onto which a material of interest can be deposited [2], but the substrate may also need to fulfill a variety of additional requirements for specialized studies. Key properties of the substrate may include optical transparency, thermal stability, mechanical strength, and chemical stability, among others. Thus, the selection of an appropriate substrate can be challenging, as an experimentalist must consider many different requirements for the substrate material, and the relevant properties will vary depending on the testing parameters.

The electrochemical reactivity of the substrate is a key criterion which is particularly important when choosing a substrate for electrochemical applications. In most cases, an inert substrate that exhibits no electrochemical activity under the testing conditions is preferred. Such a substrate facilitates a straightforward analysis because all electrochemical features can be attributed to the active material. In practice, this ideal is never perfectly attained, as the substrate almost always contributes some electrochemical features through capacitance, surface phase changes, or background electrocatalysis [1], [2]. In some cases, the substrate can also modify the properties of the electrocatalyst or photoelectrode material [3], [4]. This type of interaction can be either beneficial or detrimental to the performance of the system, and as these interactions can be difficult to predict and control, they are not routinely desired for evaluating electrocatalysts or photoelectrodes. Thus, for the majority of electrocatalyst or photoelectrode evaluations, the best strategy is to choose a substrate which approximates an ideal inert support as closely as possible under the given testing conditions. Assessing the electrochemical reactivity of a substrate, however, can be a challenge in its own right because the observed behavior depends not only on the properties of the substrate, but also on the electrolyte, voltage range, temperature, gas purge, and other testing conditions [5].

The difficulties associated with selecting an appropriate substrate are confounded by the wide array of potential substrate materials and the lack of systematic published data aimed at aiding in the selection. The electrochemical reactivity of many individual candidate substrate materials such as indium tin oxide and gold has been studied extensively [6]–[14], but these studies have been performed under widely different conditions, and applying these data with the aim of selecting an appropriate substrate is not straightforward. In contrast, there are few reports about the electrochemical reactivity of many other substrate materials such as aluminum-doped zinc oxide. While there have been a few efforts to address this issue over the past several decades [15], to the best of our knowledge, there has been no comprehensive and systematic experimental study of electrochemical substrate materials with the aim of developing guidelines for appropriate substrate selection.

In this work, we employ a consistent experimental methodology to examine the electrochemical reactivity and stability of several transparent and opaque conductive materials that are frequently used as substrates in the evaluation of electrocatalyst and photoelectrode materials. We evaluate three transparent conducting oxide substrates (indium tin oxide [13], [16]–[22], fluorine-doped tin oxide [17], [19], [22]–[24], and aluminum-doped zinc oxide [25]–[29]) and four opaque substrates (gold [6]–[8], [11], [30]–[39], stainless steel 304 [5], [40]–[48], glassy carbon [15], [49]–[69], and highly oriented pyrolytic graphite [70]–[80]). We use testing parameters that approximate the conditions commonly employed in the evaluation of electrocatalyst and photoelectrode materials. Using cyclic voltammetry with a progressively increased scan range, we evaluate the electrochemical reactivity of each substrate in acidic, neutral, and basic aqueous electrolyte. These data reveal the potential window over which each substrate exhibits minimal electrochemical features. These results provide useful insights into the behavior of these materials and serve as a starting point for the selection of appropriate substrate materials for evaluating novel electrocatalysts and photoelectrodes.

## Experimental Methods

### Substrate Preparation

All substrates were rigorously cleaned prior to testing by following the standard practice for each particular material type. An excellent review of substrates and the appropriate preparation conditions is available elsewhere [1]. Indium tin oxide (ITO, Delta Technologies, 150–200 nm on aluminosilicate glass, 4–10 Ω/sq), fluorine-doped tin oxide (FTO, Hartford Glass, ∼600 nm on soda lime glass, 6 Ω/sq), and aluminum-doped zinc oxide (AZO, Advanced Film Services, 1.3 µm on soda lime glass, 6 Ω/sq) were cleaned by sequential sonication for 30 min each in the following solvents: soapy water, acetone, isopropanol, and Millipore water. The substrates were then dried in ambient air. Gold foils (Alfa Aesar, 0.127 mm, 99.99% metals basis) were hydrogen flame annealed then soaked in 30% nitric acid overnight. After rinsing in Millipore water, the hydrogen flame annealing process was repeated. Stainless steel foils (SS, Alfa Aesar, 0.1 mm, SS 304) were soaked in 0.5 M sulfuric acid for cleaning followed by rinsing with Millipore water. The glassy carbon (GC) disk electrodes were prepared from 200 mm long glassy carbon rods (SigradurG, HTW Hochtemperatur-Werkstoffe GmbH, 5 mm diameter). These rods were processed by the Stanford University crystal shop to prepare 4 mm long pieces with the top side polished to a surface RMS roughness of less than 50 nm. The glassy carbon pieces were sonicated in Millipore water for 15 minutes prior to electrochemical characterization. The highly oriented pyrolytic graphite (HOPG, SPI Supplies, 1 cm×1 cm) surface was prepared by freshly cleaving the crystal followed by a 3 min anodization in 0.2 M phosphate buffer (pH 7.2) at 1.65 V vs. Ag/AgCl. Such a pre-treatment is common when using HOPG to roughen the surface and provide edge sites for electrocatalyst deposition [81], [82].

### Electrochemical Characterization

Electrochemical testing of all materials except for glassy carbon was carried out in a polytetrafluoroethylene (PTFE) compression cell setup in a standard three electrode configuration. The diameter of the exposed substrate was 8 mm corresponding to an area of 0.503 cm^2^. Glassy carbon testing was performed in a rotating disk electrode configuration (Pine Instruments) but without rotation. The electrode diameter was 5 mm resulting in an area of 0.196 cm^2^. For all testing, a Ag/AgCl (4 M KCl) reference electrode and a Pt wire or mesh counter electrode were used. The reference electrode was regularly calibrated to the reversible hydrogen electrode (RHE) in each electrolyte and the data shifted accordingly. A potentiostat (Bio-Logic VMP3 or VSP) was used for potential control and data acquisition. The pH values of the three freshly prepared electrolyte solutions were measured as follows: 0.1 M H_2_SO_4_ (pH 1.0), 0.1 M NaAc (pH 7.2–7.8), and 0.1 M NaOH (pH 13.0). All solutions were prepared from reagent grade chemicals without further purification. The electrolyte was purged with N_2_ throughout testing via a glass dispersion frit. Potentio-electrochemical impedance spectroscopy (PEIS) was employed to measure the series resistance at open circuit and compensate for 85% of the iR-drop in situ [83]. In situ compensation of 100% of the iR-drop is not possible as it can lead to instability in potentiostat control [2]. A mathematical correction for the remaining 15% was applied in post-processing of the data by subtracting 15% of the series resistance multiplied by the current (i.e. the iR-drop) from the potential at each point on the cyclic voltammogram [84].

### Testing Methodology

A progressive scan methodology was applied to examine the cathodic and anodic inert potential windows of the seven substrates in each of the three electrolytes (acid, neutral, and base). An intermediary starting potential close to 0.35 V vs. RHE (0.10 V vs. Ag/AgCl in acid, −0.25 V vs. Ag/AgCl in neutral, and −0.60 V vs. Ag/AgCl in base) was selected to delimit the cathodic and anodic testing windows. For anodic scans, the potential was swept 100 mV positive of the starting potential at a rate of 25 mV/s and then swept back. This scan rate is within the common range for electrocatalyst and photoelectrode studies [85], [86]. This cyclic scan was repeated 1 to 3 times, depending on whether any redox features were observed. If a new feature emerged, the scan was repeated within the same window to observe if it changed over time or was stable. The anodic vertex potential was increased in 100 mV increments in this manner until a current density greater than 2 mA/cm^2^ was achieved. Using a fresh substrate, the process was repeated in the cathodic direction, again increasing the scan range in increments of 100 mV until a current density of −2 mA/cm^2^ was reached. Anodic and cathodic scans were performed in each of the three electrolytes such that six individual samples of each substrate material make up a complete set of data. While up to 2 mA/cm^2^ of current was drawn to facilitate identifying features (e.g. catalytic water oxidation or reduction vs. a peak arising from redox cycling of an element), 50 µA/cm^2^ above the baseline capacitance was defined as the cut-off at which a substrate is no longer considered inert for the purposes of this study. Inertness refers here to whether there are any redox features or background activity whereas stability refers to whether or not there are changes in the composition or properties of the electrode over time, either from being immersed in the electrolyte or from applying a potential.

### Molybdenum Sulfide Hydrogen Evolution Catalysis

An amorphous molybdenum sulfide hydrogen evolution catalyst was synthesized following a previously reported procedure [87]. The catalyst was dispersed in isopropanol and drop cast onto a clean FTO substrate with a mass loading of approximately 0.2 mg/cm^2^. The activity of this catalyst was measured via potential cycling from 0.15 to −0.30 V vs. RHE in 0.1 M H_2_SO_4_ electrolyte. For comparison, a bare FTO substrate was also cycled over a single large potential window of −0.85 to 2.85 V vs. RHE beginning at a potential of 0.35 V vs. RHE with the first sweep in the cathodic direction.

## Results and Discussion

### Testing Methodology

**Figure 1:**
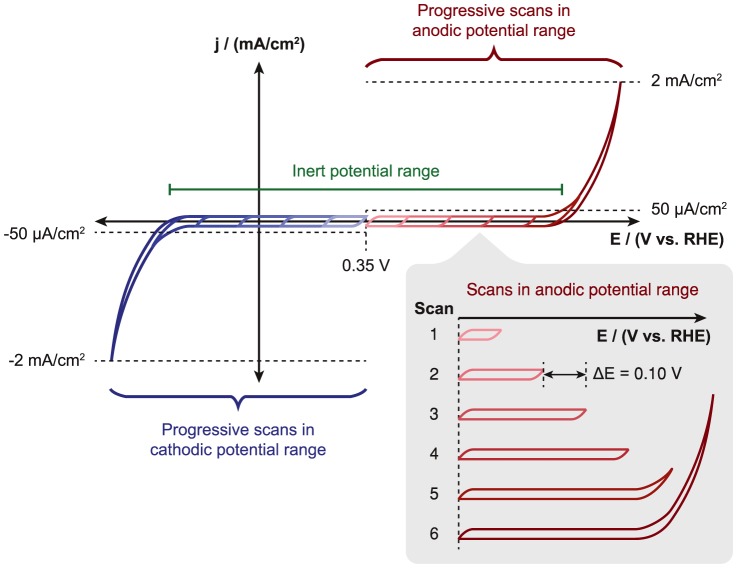
Testing methodology.

The testing procedure outlined in Section 2.3 is shown graphically in Figure 1. The progressive scanning technique employed in this study has many benefits. Most importantly, it facilitates the correlation of oxidative features with the corresponding reductive process and vice versa. Each substrate has a finite inert potential window under a given set of conditions, and progressive scanning allows accurate determination of this window as the substrate is not irreversibly degraded at the outset from scanning to very positive or negative potentials.

**Figure 2:**
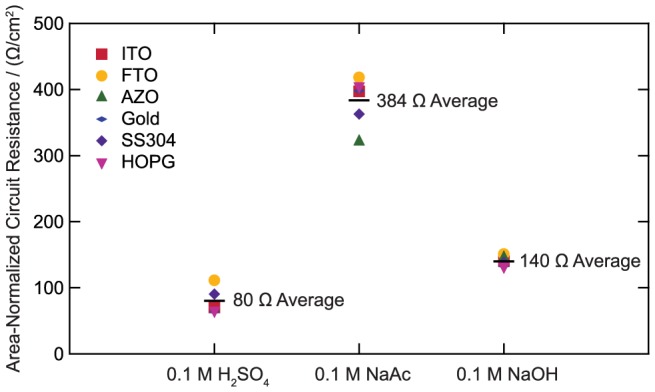
Area-normalized circuit resistances.

The measured circuit resistances for six of the substrates are shown in Figure 2 as area-normalized values. These values show a strong correlation to the type of electrolyte. While the FTO consistently exhibits a slightly higher circuit resistance, the measured value is primarily dictated by the mobility and concentration of current-carrying ions in the solution [2]. The circuit resistance also depends on the distance between the working electrode and reference electrode. While this distance was approximately constant for all the electrodes tested in a compression cell setup, it was larger for glassy carbon, which was tested in a rotating disk electrode configuration. This resulted in higher area-normalized circuit resistance values for GC. Since the substrates themselves represent a minimal contribution to the circuit resistance, the data was voltage-compensated for 100% of the resistance (85% in situ and 15% in post-processing). It is important to note that the conductivity of some substrates, such as the transparent conductive oxides FTO and ITO, is heavily influenced by heat treatment conditions. Both the temperature and annealing atmosphere can influence the doping density and thus resulting conductivity [88]. The substrates in this study were all used as-received (except for the annealed gold foil and anodized HOPG) and no major changes were expected. When studying heat treated supported electrocatalysts, care must be taken to ensure any drop or rise in performance is due to intrinsic activity of the catalyst rather than a change in substrate conductivity.

### Transparent Substrates

Transparent conducting oxides (TCOs) are typically degenerately-doped wide band gap (>3 eV) semiconductors [16], [17], [89]. For many common n-type substrates, free electrons in the conduction band are generated by oxygen vacancies or substitution of the host metal by higher valency metal atoms (e.g. Sn in Sn:In_2_O_3_ or Al in Al:ZnO) [90]. TCOs are employed commercially in a number of solid-state devices and are also used extensively as substrates for electrochemical studies [89]. Their high degree of transparency facilitates optical absorption measurements of catalysts, enables spectroelectrochemical studies, and permits both front and back-side illumination for the study of photoelectrodes. The exact synthetic route can affect their transmittance and conductivity significantly, so substrates should be selected with the appropriate specifications for a given application [23].

### Indium Tin Oxide

Indium tin oxide, the most prevalent TCO substrate, consists of a solid solution of In_2_O_3_ and SnO_2_ with 5–10 atomic % Sn [17]. A thin layer is typically sputter-coated on an inert glass support [18]. It is employed commercially in a number of products including displays, coatings, and solar cells [19]. Further widespread use may be limited by the cost and scarcity of indium [16], [91].

**Figure 3:**
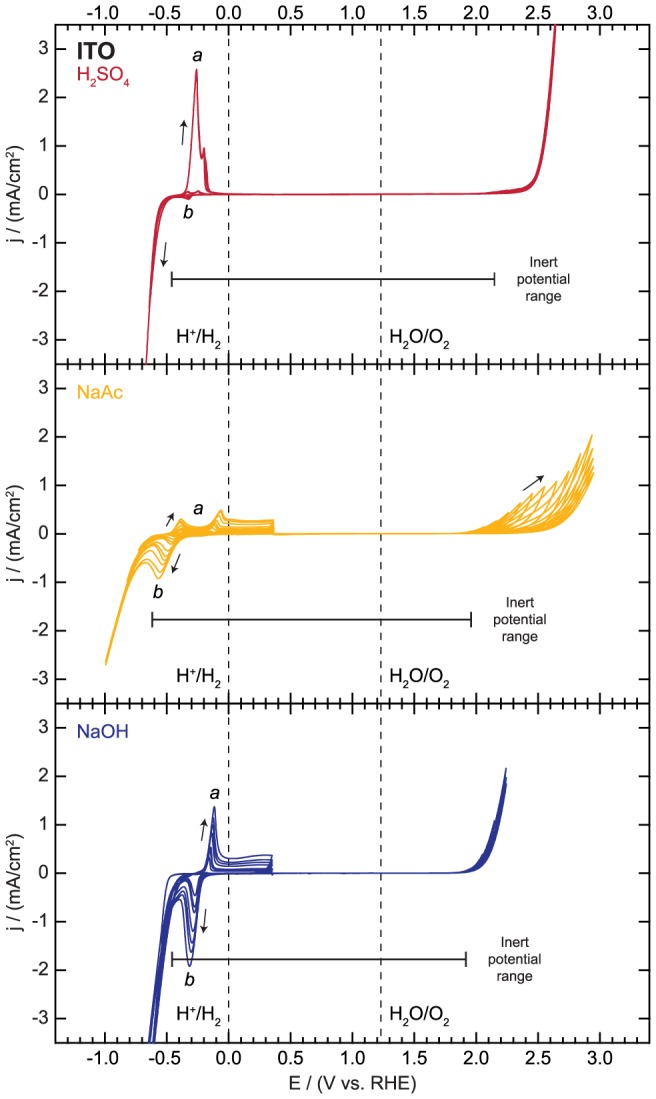
Electrochemical activity and inert potential range for indium tin oxide (ITO).

The results of the progressive electrochemical cycling of ITO are shown in Figure 3. The sweeps in the cathodic region show several significant redox features apart from the hydrogen evolution reaction (HER) in all three electrolytes. The oxidative features (denoted *a* in Figure 3) appear only when sweeping towards positive potentials after a reductive current is drawn upon scanning to potentials of −0.55, −0.48, and −0.45 V vs. RHE in H_2_SO_4_, NaAc, and NaOH, respectively. On subsequent cycles, a corresponding reduction peak (denoted *b* in Figure 3) appears prior to the onset of hydrogen evolution. All features grow as the progressive scanning is extended to more negative potentials. These redox features are attributed to reduction of Sn and In to lower valence or metallic states and subsequent partial reoxidation/rereduction [1], [13], [20]. Previous work has shown by chemical analysis that these changes extend at least several nm deep [20]. It is also possible that some oxidized ions dissolve into solution and can plate back onto the electrode on subsequent cycles. This cycling of the redox states leads to irreversible changes to the electrode which degrade its electrical and optical properties. During cycling or when held at potentials more negative than −0.55 V vs. RHE, the ITO electrode is observed to turn gray in color due to the metal cation reduction, resulting in a significant decrease in its transparency.

In contrast to the cathodic region, sweeps in the anodic region are relatively featureless. The only significant current arises from water oxidation through the oxygen evolution reaction (OER), with the earliest onset at 1.92 V vs. RHE in NaOH and the latest onset at 2.15 V vs. RHE in H_2_SO_4_. While these potential limits may appear to make ITO an ideal substrate for testing catalysts at positive potentials, there is a slow degradation of the substrate. There is a progressive decrease in water oxidation current in the NaAc electrolyte upon cycling. A similar trend, though less pronounced, is also observed in both H_2_SO_4_ and NaOH. During anodic polarization, In-O and Sn-O bonds are broken as lattice oxygen (O^2−^) is oxidized, resulting in O_2_ evolution and dissolution of In and Sn [21]. Elemental analysis of the electrolyte confirms the presence of both In and Sn but with a higher than expected In:Sn ratio. Under certain conditions, stable SnO_2_ crystallites can reform on the surface [21]. The net result is an increase in the surface roughness but also an increase in the resistance of the substrate due to the SnO_2_ passivating layer. The rate of dissolution is strongly influenced by the nature of the ions present in the electrolyte, with ions capable of better solvating In and Sn, such as Cl^−^, potentially accelerating the process.

After sweeps in both the cathodic and anodic regions in H_2_SO_4_, a clear color change of the ITO film in the tested area is visually observed. According to the Pourbaix diagram, In_2_O_3_ is unstable at all potentials at pH = 1 so it is likely dissolving into solution [22]. ITO is therefore an unsuitable substrate in this electrolyte.

In summary, while ITO has an electrochemically inert potential window extending from −0.46 to 2.15 V vs. RHE in 0.1 M H_2_SO_4_, it is chemically unstable in this electrolyte, and therefore not recommended. ITO is an appropriate substrate for electrochemical studies in 0.1 M NaAc and 0.1 M NaOH across a wide region extending between −0.62 to 1.96 V vs. RHE and −0.45 to 1.92 V vs. RHE, respectively, where the reduction of metals atoms and significant water oxidation current can be avoided. However, it should not be employed for extended stability tests if the supported film does not completely cover the ITO surface. The length of time (i.e. minutes, hours, or days) over which the ITO will be sufficiently stable depends on the electrolyte concentration, the potential range, and the extent of coverage of the catalyst. Otherwise, slow leaching of Sn and In degrades the electrical properties and can lead to complete failure of the electrode.

### Fluorine-doped Tin Oxide

Fluorine-doped tin oxide is a SnO_2_-based wide band gap semiconductor with fluorine doping on the order of 5×10^20^–10^21^ cm^−3^ [23]. FTO also has commercial applications, primarily in energy efficient windows [17]. While it can be more challenging to synthesize, it has better mechanical and chemical durability than other TCOs and is less expensive [19], [24].

**Figure 4:**
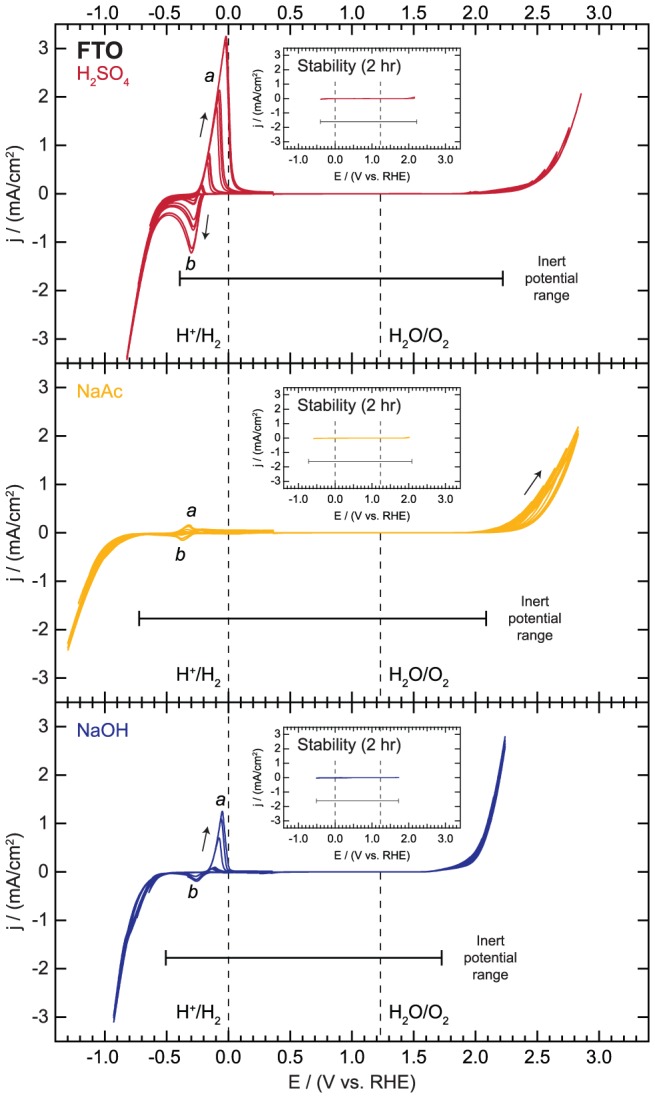
Electrochemical activity and inert potential range for fluorine-doped tin oxide (FTO). Inset for each electrolyte shows polarization curves for continuous cycling within the inert potential range for a period of 2 hours.

The progressive cycling of FTO is shown in Figure 4. The features associated with the cycling of FTO in the cathodic region are very similar to those of ITO. In H_2_SO_4_, the sweep is featureless until a small reductive current is observed at a potential of −0.39 V vs. RHE. Upon sweeping back to positive potentials, an oxidative peak (denoted *a* in Figure 4) appears which also scales with the amount of reductive current drawn during progressive cycling to more negative potentials. The reductive feature (denoted *b* in Figure 4) only grows in after the oxidative peak is observed. These redox features are once again attributed to changes in the oxidation state of Sn. A similar evolution of peaks is observed in the case of the NaAc and NaOH electrolytes but at more negative potentials. Hydrogen evolution currents of nearly 1 mA/cm^2^ at potentials of −1.19 and −0.85 V vs. RHE in NaAc and NaOH, respectively, are drawn before any Sn redox features appear.

There are no significant features in the progressive cycling in the anodic region other than catalytic water oxidation, which onsets at potentials of 2.22, 2.09, and 1.73 V vs. RHE in H_2_SO_4_, NaAc, and NaOH, respectively. There is a slight decrease, most pronounced in NaAc, in the water oxidation current with cycling. The origin of this decrease is currently unknown. The leaching of Sn from SnO_2_ has not been reported on long time scales. Unlike the In_2_O_3_ in ITO, the Sn is already present as a stable SnO_2_ phase at all pHs and electrode potentials tested here [22]. While there is very little literature on the long-term stability of FTO at different pHs, our experiments confirm that FTO substrates are stable for at least two hours. We cycled the potential of the FTO substrates within the stability bounds determined herein for two hours and found no evidence of any new electrochemical features, as shown in the Figure 4 inset panels. The inert potential range decreased by 1% or less in each electrolyte after the stability cycling.

In summary, FTO is a suitable substrate for use in all three electrolytes over a wide range of potentials. The inert region, bounded mainly by the onsets of hydrogen and oxygen evolution, corresponds to potentials between −0.39 and 2.22 V vs. RHE, −0.72 and 2.09 V vs. RHE, and −0.51 and 1.73 V vs. RHE in 0.1 M H_2_SO_4_, 0.1 M NaAc, and 0.1 M NaOH, respectively.

### Aluminum-Doped Zinc Oxide

Aluminum-doped zinc oxide is another low-cost TCO option which exhibits good optical transmission. The level of Al doping is typically less than 5% [25]. Properties which distinguish AZO from other TCOs are its resistance to hydrogen-rich plasmas and a lower work function which makes it more suitable than ITO or FTO for use as a cathode support [26]. It is however highly unstable in acid. We were unable to test the AZO in H_2_SO_4_ because the material dissolved immediately upon immersion in the electrolyte.

**Figure 5:**
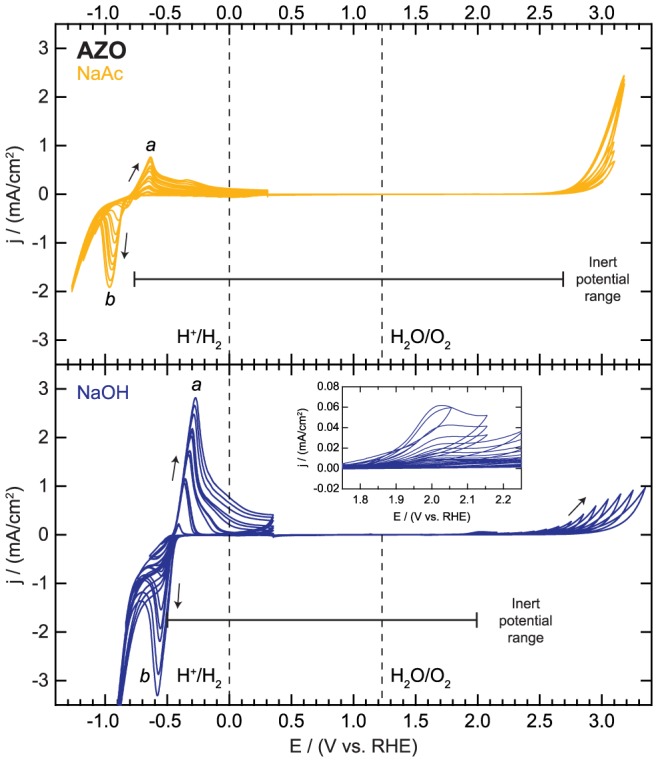
Electrochemical activity and inert potential range for aluminum-doped zinc oxide (AZO).

The results of cycling in the other two electrolytes are shown in Figure 5. The AZO exhibits a similar pattern to both ITO and FTO in terms of redox features appearing in the cathodic region. The baseline is flat until a potential of −0.77 or −0.50 V vs. RHE in NaAc and NaOH, respectively, when a small reductive current begins. The oxidation feature (denoted *a* in Figure 5) then appears on the sweep to more positive potentials followed by the reduction feature (denoted *b* in Figure 5) on the subsequent sweep in the cathodic direction. These features are attributed to the cycling of the Zn oxidation state. Previous work has shown that Zn^2+^ is reduced to metallic Zn and subsequent oxidation/reduction occurs via various soluble zincate complex ions (e.g. Zn(OH)_2_, Zn(OH)_3_^−^, Zn(OH)_4_^2−^) [27], [28].

The primary feature of the progressive cycling in the anodic region is catalytic water oxidation, though there is a small (<100 µA/cm^2^) oxidative pre-feature in the NaOH at 2.00 V vs. RHE which decays rapidly after the first few cycles (shown in Figure 5 inset). The activity for water oxidation is highly unstable in both electrolytes, requiring a progressively more positive potential to draw any current. In fact, the target current of 2 mA/cm^2^ was not attained in NaOH due to the current falloff. ZnO is not stable in aqueous solutions at any pH and the rate of dissolution is rapid for a pH≤5 or pH≥11 [29]. Even at neutral pH, the electrode slowly corrodes to form Zn^2+^, ZnOH^+^, Zn(OH)_3_^−^, and Zn(OH)_4_^2−^ species which eventually leads to catastrophic failure.

In summary, the inert potential region corresponds to potentials between −0.77 and 2.69 V vs. RHE in 0.1 M NaAc and −0.50 and 1.99 V vs. RHE in 0.1 M NaOH. However, AZO is not suitable as a substrate in any electrolyte on any time scale relevant to electrochemical cycling, except possibly if the catalyst or photoelectrocatalyst forms a truly conformal, pin-hole free layer on the substrate to protect it. If this were the case, its primary advantages would be a low sheet resistance and a low work function.

### Opaque Substrates

Opaque substrate materials are appropriate for evaluating electrocatalysts and photoelectrodes when back-side illumination or transmission experiments are not necessary. While there are many metallic conductors that might be appropriate choices for electrochemical substrate materials, we have chosen to analyze several in particular that may be appropriate under different experimental conditions.

### Gold

Gold is an appropriate electrode material to consider because of its very low chemical reactivity. Gold has been called “the noblest of all the metals” [30] because of its chemical inertness. The galvanic potential of gold is very high [31], which means that it is not susceptible to corrosion. Additionally, the electrochemical behavior of gold has been studied extensively [6]–[8], [11], [32]–[39], and thus it may be easier to predict and understand the behavior of a gold electrode. A key drawback of gold is its price of over $40 per gram as of June 2014 [92], which may make gold an impractical choice when a large amount of substrate is required.

**Figure 6:**
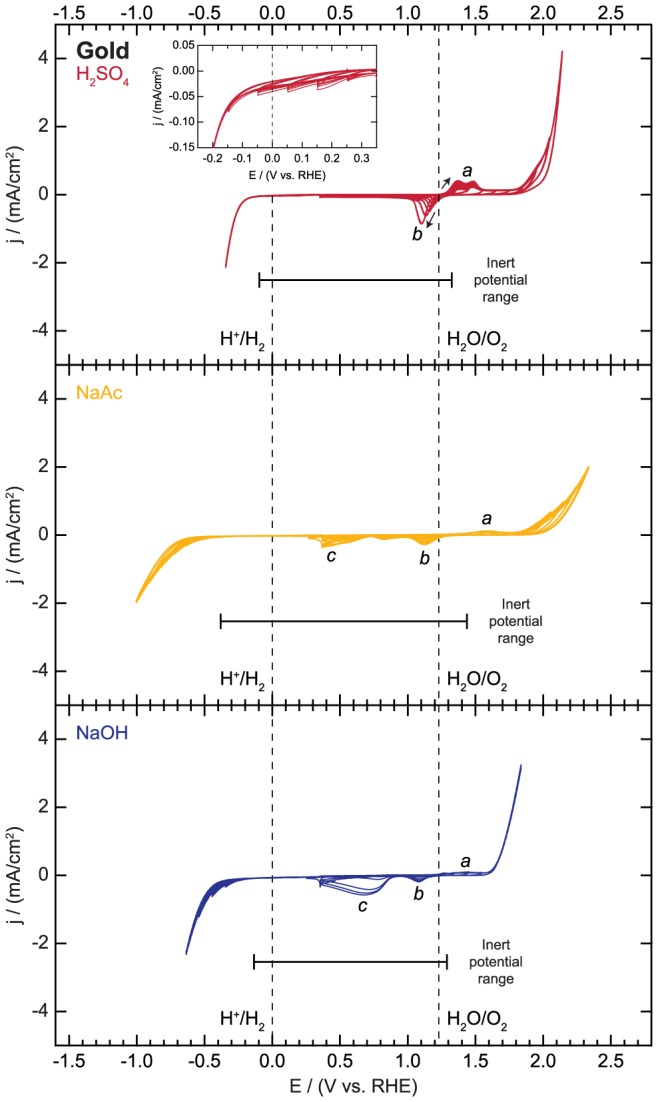
Electrochemical activity and inert potential range for gold.

The results of the electrochemical reactivity tests on gold are presented in Figure 6. The sweeps in the cathodic region show few features. In each electrolyte, the only reaction observed is hydrogen evolution. The gold surface is most active for the HER in H_2_SO_4_. In this electrolyte, the electrochemically inert potential range extends to −0.10 V vs. RHE (shown in Figure 6 inset), and the HER activity of the gold surface remains constant over progressive cathodic cycles. Note in the inset the slight decrease in the baseline current at potentials positive of the reversible potential for hydrogen evolution; the origin of this decay is unknown but was not investigated due to the very small magnitude of the change. The HER activity of the gold surface is lower in both NaAc and NaOH solutions, so the region with no electrochemical features is larger. In these solutions, the HER onset shifts to slightly more negative potentials with continued cycling, showing that the HER activity of the gold surface decreases slightly, possibly due to surface restructuring.

The sweeps in the anodic region on the gold surface are more complex. In each electrolyte, anodic oxidation of the gold surface precedes the oxygen evolution reaction. Detailed explanations of the gold oxidation features are provided elsewhere [6]–[8], [11], [32]–[35], [37]–[39]. A series of oxidative features (denoted *a* in Figure 6) corresponding to the adsorption of OH^−^ groups and the initial oxidation of the gold surface is observed between 1.30 and 1.60 V vs. RHE in each electrolyte. These features may also arise from the adsorption of other anions such as the sulfate ions in the H_2_SO_4_ electrolyte [39]. The two peaks observed near 1.40 and 1.50 V vs. RHE in H_2_SO_4_ grow in size with continued cycling, corresponding to increasingly rapid surface oxidation on each subsequent cycle, reaching a maximum current density of around 0.4 mA/cm^2^, while these oxidation features only grow to around 0.15 mA/cm^2^ in NaAc and NaOH. In each electrolyte, the oxidative features are accompanied by corresponding reductive peaks (denoted *b* in Figure 6) appearing between 1.00 and 1.20 V vs. RHE on the reverse cycle. These features correspond to the desorption of OH^−^ or SO_4_^2−^ ions or reduction of the gold oxide surface [37], [39]. At more anodic potentials, the gold surface catalyzes the OER. After oxygen is evolved, current (denoted *c* in Figure 6) corresponding to oxygen reduction is observed on the reverse sweep due to incomplete removal of O_2_ from the surface by the N_2_ purging. This feature is especially apparent in NaOH electrolyte, with an oxygen reduction feature appearing prominently at about 0.70 V vs. RHE.

In summary, the inert potential region corresponds to potentials between −0.10 and 1.33 V vs. RHE, −0.38 and 1.44 V vs. RHE, and −0.14 and 1.29 V vs. RHE in 0.1 M H_2_SO_4_, 0.1 M NaAc, and 0.1 M NaOH, respectively. Gold electrodes may be appropriate for evaluating some materials at potentials less than 0.00 V vs. RHE due to the lack of any substantial electrochemical features other than the HER, though this reaction could interfere with measurements at large negative potentials, especially in acidic electrolyte. Gold may not be an ideal substrate material for studies requiring potentials higher than ca. 1.30 V vs. RHE due to the features corresponding to gold oxidation that are observed in each electrolyte and the gold's reasonably high activity for oxygen evolution, especially in alkaline electrolyte.

### Stainless Steel

Similar to gold, stainless steel is known to be chemically inert and resistant to corrosion in many types of electrolytes [5]. Unlike gold, which is inert due to its high galvanic potential, the corrosion resistance of stainless steel is conferred by its passivating, chromium-rich native oxide [40], [41]. Stainless steel is much less expensive than gold, an attractive feature for studies that require very large electrodes or many samples. Stainless steel also has excellent mechanical properties [42]. However, since stainless steel is an alloy that contains many elements such as iron, chromium, nickel, and carbon [42], the complexity of this material increases the risk of contamination or undesirable side reactions. This difficulty is amplified by the large number of available stainless steel varieties, which may have quite different electrochemical properties [5], [40]. We chose to evaluate SS 304 because it is the most widely used type of stainless steel and is considered to exhibit excellent corrosion resistance [42], [43]. SS 304 is composed of iron alloyed with 18–20% chromium, 8–12% nickel, up to 2% manganese, and small amounts of carbon, phosphorus, sulfur, silicon, and nitrogen [42].

**Figure 7:**
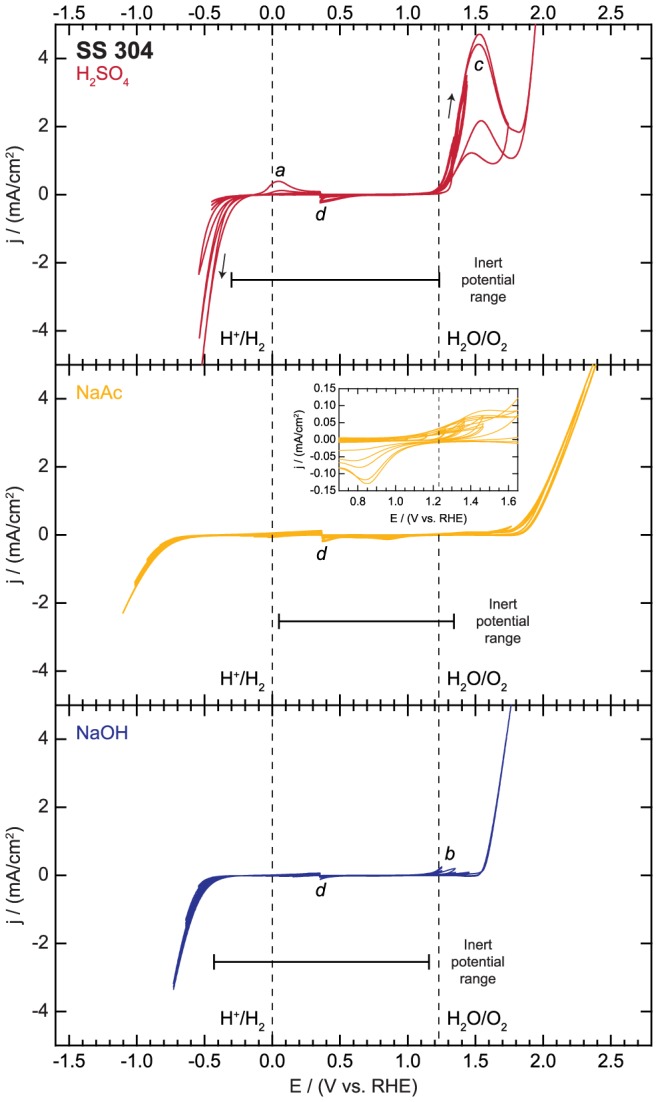
Electrochemical activity and inert potential range for stainless steel 304 (SS 304).

The electrochemical reactivity data for our stainless steel 304 samples are presented in Figure 7. In the sweeps in the cathodic region, no features are observed until the onset of the HER at −0.30 V vs. RHE in H_2_SO_4_. The HER activity increases substantially with cycling, possibly due to surface restructuring and/or the reduction of the surface oxide, and an oxidative peak (denoted *a* in Figure 7) appears in the final several cycles. This feature has previously been attributed to the oxidation of hydrogen atoms absorbed within the stainless steel during the HER [44]. In NaAc, we observe a small reductive feature at −0.04 V vs RHE that likely corresponds to native oxide reduction. This feature decreases in size with repeated cycling, but limits the cathodic inert range to 0.05 V vs RHE. The only other reductive feature corresponds to the HER, which is first observed at approximately −0.73 V vs. RHE. The HER activity again increases slightly with cycling. In NaOH, we observe an oxidation/reduction couple with peaks at 0.26 and 0.00 V vs. RHE and very small currents of less than 10 µA/cm^2^. This couple likely corresponds to nickel oxidation and reduction [45]. The inert potential range extends to −0.43 V vs. RHE, where the onset of the HER is initially observed. The HER activity increases slightly with repeated cycling.

In the sweeps in the anodic region, the observed features generally correspond to either the OER or metal oxidation/dissolution [40], [41]. In H_2_SO_4_, a large oxidative peak (denoted *c* in Figure 7) is observed. Prior studies of stainless steel corrosion have suggested that this feature arises from the dissolution of the chromium-rich native oxide in the transpassive (i.e. highly anodic) potential regime [46], [47]. The OER wave begins around 1.85 V vs. RHE and may be accompanied by oxidative metal dissolution. In NaAc, we observe a couple with small oxidation and reduction peaks at 1.45 and 0.80 V vs. RHE, respectively (shown in Figure 7 inset). This couple has peak currents less than 100 µA/cm^2^, and likely corresponds to a reversible change in the oxidation state of the native chromium oxide. The inert potential range extends to 1.34 V vs. RHE, and the onset of OER is observed at around 1.65 V vs. RHE. Some oxidative metal dissolution may also occur in the highly anodic potential regime. In NaOH, an oxidative feature (denoted *b* in Figure 7) is observed with no corresponding reductive peak, and the size of this feature decreases with cycling. This feature may be associated with either surface oxidation or the dissolution of the native oxide. Due to the size of this peak, the anodic inert potential limit occurs at 1.16 V vs. RHE, but it may be possible to use SS 304 at more positive potentials if this initial oxidative feature is unimportant for a given application. The onset of the OER is observed at 1.55 V vs. RHE. Some oxidative dissolution of iron, nickel, or other metals may also occur in this regime [40], [41]. On the final sweeps in the negative direction after oxygen has been evolved in each electrolyte, a small reductive feature is observed near 0.35 V vs. RHE (denoted *d* in Figure 7) arising from the reduction of oxygen that remains near the electrode.

In summary, the inert potential region corresponds to potentials between −0.30 and 1.23 V vs. RHE, 0.05 and 1.34 V vs. RHE, and −0.43 and 1.16 V vs. RHE in 0.1 M H_2_SO_4_, 0.1 M NaAc, and 0.1 M NaOH, respectively. Stainless steel may be an appropriate substrate for use in alkaline electrolytes, where few features aside from the HER and OER are observed. It may also be a good choice for specialty applications that require high mechanical strength or a large number of metal substrates. Otherwise, the relatively small inert potential range of this material in acidic and basic electrolyte may make this substrate a less than ideal choice for many studies. We consider it especially important for researchers who wish to use stainless steel electrodes to conduct their own experiments to determine the inert potential range, because the electrochemical behavior of stainless steel may change substantially depending on the details of the exact starting material and the experimental procedures [5]. For example, electrolytes containing chloride ions may result in increased corrosion of stainless steel [40]. Additionally, as observed in the H_2_SO_4_ electrolyte, cathodic polarization and hydrogen evolution can lead to hydrogen absorption, which could change the oxidative behavior of the electrode if a broad potential range is required [44], [48].

### Glassy Carbon

Glassy carbon has been widely used as an electrode material since its discovery in 1962 [49], [50]. This material consists of tangled graphite nanoribbons and possesses no long-range atomic ordering [15], [51]. Glassy carbon is an ideal substrate for many electrochemical studies because it is chemically stable and electrochemically inert in a large potential window [49], [51]–[53]. Unlike many other conductive carbon materials, glassy carbon is generally impermeable to gases and can be polished to a mirror finish [15], [52]. Additionally, glassy carbon can be readily obtained in disk form for use with a rotating disk apparatus. Finally, glassy carbon may be useful for studies involving spectroscopic characterization techniques because its single element composition typically produces a clean background signal. These features have made glassy carbon a convenient choice for many studies of electrocatalyst materials.

**Figure 8:**
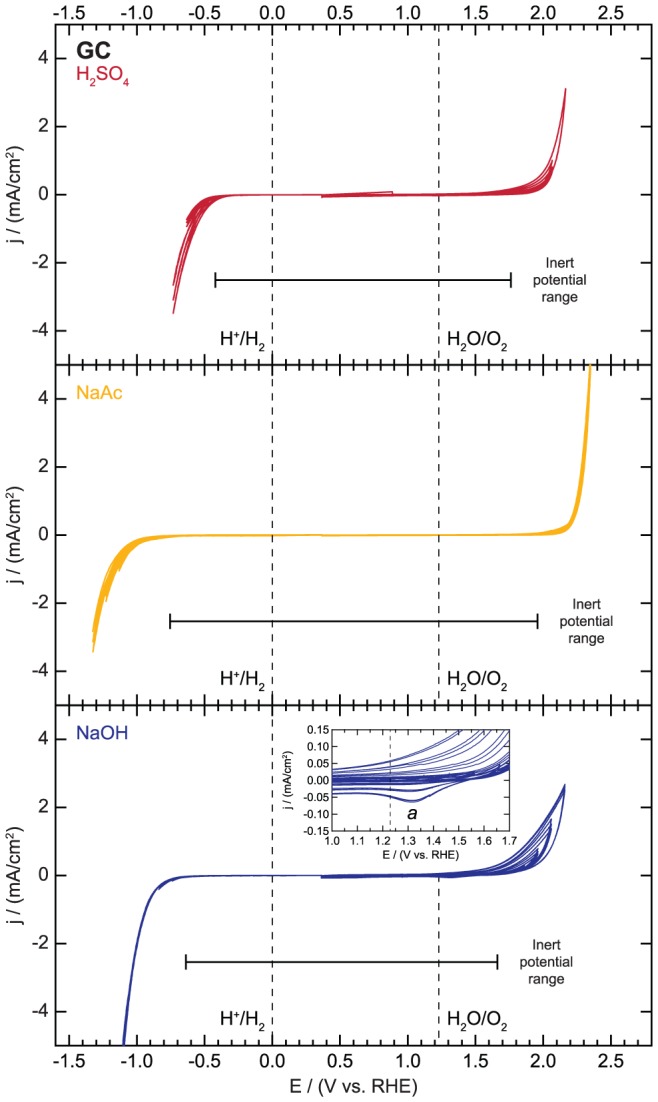
Electrochemical activity and inert potential range for glassy carbon (GC).

The main electrochemical reactions expected on glassy carbon are the electrolyte decomposition reactions (i.e. the HER and OER), ion adsorption/desorption, and oxidation/reduction of the glassy carbon surface [15], [49], [51], [54]. As shown in Figure 8, the HER is the main electrochemical feature observed in the cathodic region and limits the inert potential range for each electrolyte. In H_2_SO_4_ and NaOH, the HER activity of the glassy carbon increases slightly with potential cycling, possibly due to a reduction of any oxidized surface species or removal of surface impurities. In contrast, the HER activity decreases slightly with potential cycling in the NaAc.

The anodic stability window is limited by the onset of the OER in H_2_SO_4_, and the OER activity increases slightly with cycling. Likewise, in NaAc, the only feature is the OER wave, but the activity does not change with potential cycling. In NaOH electrolyte, the onset of oxidative current is initially observed at 1.66 V vs. RHE. With subsequent potential cycles, the onset of the oxidative reaction shifts to a potential of around 1.30 V vs. RHE where a small reductive peak appears as well (denoted *a* in Figure 8 inset). The combination of this reductive peak and the large hysteresis in the oxidative potential sweeps suggests that the oxidative features correspond to a combination of oxygen evolution and oxidation of the glassy carbon to produce carbon dioxide or oxidized surface species [15], [52], [55]. The magnitude of the oxidative peak might be increasing due to an increase in the rates of these oxidative reactions as the surface is cleaned or roughened by the repeated formation and reduction of an oxide layer. It is also possible that as the glassy carbon surface is oxidized, the non-Faradaic capacitive current observed in this region also increases.

In summary, the inert potential region corresponds to potentials between −0.42 and 1.76 V vs. RHE, −0.76 and 1.96 V vs. RHE, and −0.64 and 1.66 V vs. RHE in 0.1 M H_2_SO_4_, 0.1 M NaAc, and 0.1 M NaOH, respectively. Glassy carbon's large inert potential window makes it an ideal substrate for studying many electrocatalyst and photoelectrode materials. However, we note that previous studies have shown that glassy carbon surfaces may possess a variety of functionalities, and that the nature of this surface can affect its electrochemical performance [15], [52], [56]–[69], thus researchers should pay careful attention to the GC surface preparation.

### Highly Oriented Pyrolytic Graphite

Highly oriented pyrolytic graphite (also called highly ordered pyrolytic graphite) is another carbon allotrope that has proven useful for many studies in electrochemistry [1], [70], [71]. It is a form of graphite made up of lamellar crystallites with a very high degree of crystallographic orientation (less than 1° angular spread in the c-axis directions) [70]. Thus, HOPG is an anisotropic material, and HOPG electrodes with either edge planes or basal planes exposed at the surface may be obtained [72]. In this study, we used basal plane HOPG (sometimes called basal plane pyrolytic graphite) [73]. A key advantage of basal plane HOPG is its very smooth surface, which typically consists of atomically-flat terraces of several hundred nanometers between step edges [1], [74], [75]. This makes HOPG a convenient support when scanning probe microscopy techniques are required [74], [76]. Similar to glassy carbon, HOPG may also be advantageous when spectroscopic techniques are necessary because of its relatively clean background signal [71]. HOPG electrodes can also be easily reused because the HOPG surface can be renewed by cleaving the electrode with a piece of tape to reveal a pristine top surface [1], [52], [71], [74].

Prior to our electrochemical analysis, we performed a pre-anodization of the freshly cleaved HOPG surface. This anodization procedure introduces edge-site defects and surface oxygen into the HOPG basal planes [1], [77]. These sites are more reactive than the basal plane sites and may improve adhesion of supported materials [81], [82]. We performed a pre-anodization in this study because this type of pre-treatment is common in other studies where HOPG was used as a substrate for the study of electrocatalyst or photoelectrode materials [81], [82].

**Figure 9:**
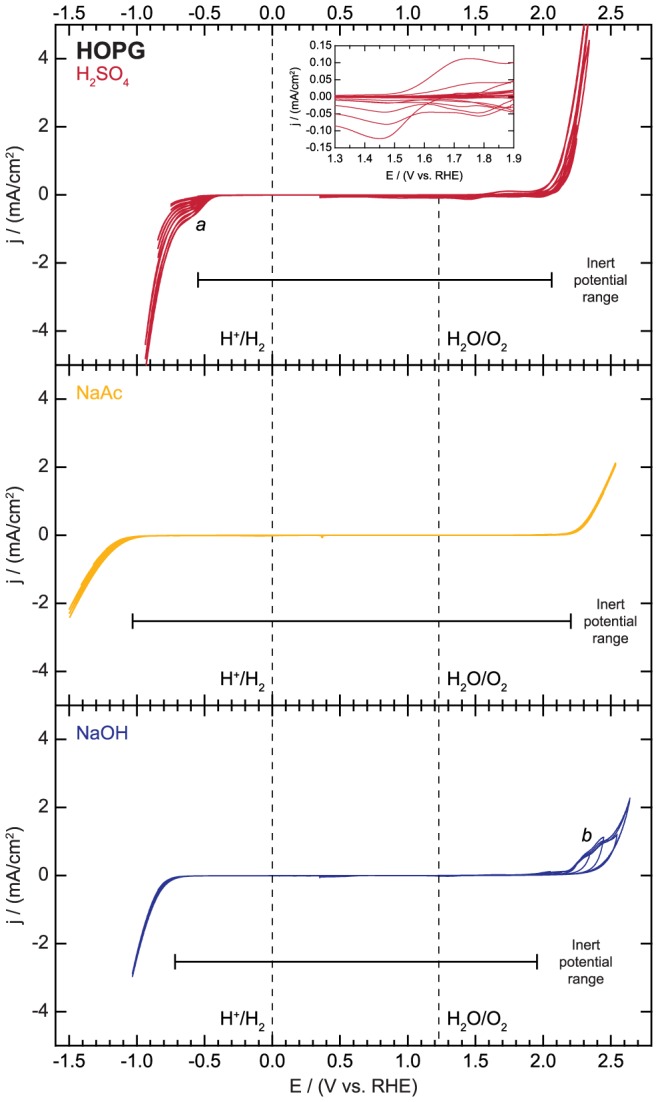
Electrochemical activity and inert potential range for highly oriented pyrolytic graphite (HOPG).

The polarization curves collected on HOPG electrodes are displayed in Figure 9. In H_2_SO_4_, the HER onset is initially observed at −0.55 V vs. RHE, but after repeated cycling, the HER activity increases and the onset shifts to a more positive potential of −0.45 V vs. RHE. A reductive shoulder (denoted *a* in Figure 9) also appears at around −0.60 V vs. RHE. The increase in HER activity with repeated cycling may be attributed to surface roughening to expose more edge sites or the reduction of surface functionalities such as ethers and hydroxyl groups [78]. The origin of the reductive shoulder is not clear, but it may also correspond to the reduction of oxidized surface groups or proton intercalation. In NaAc and NaOH, no reductive features aside from the HER are observed.

In the sweeps in the anodic region, the main electrochemical features correspond to anion intercalation, surface oxidation, and the OER [79]. In H_2_SO_4_, significant oxidative current is first observed at around 2.06 V vs. RHE. Prior studies have shown that graphite may undergo oxidation and anion intercalation in sulfuric acid in this potential regime [52], [77]. The oxidative current at the highly anodic potentials likely corresponds to a combination of these two processes along with oxygen evolution. The OER activity of the HOPG increases with potential cycling, probably due to surface cleaning or roughening. After the potential scan range is increased beyond the OER onset, a small reductive feature (shown in Figure 9 inset) is observed on the sweeps in the negative direction. This feature likely corresponds to surface oxide reduction or de-intercalation, processes that can occur within the OER potential window. In NaAc, the OER wave is the only important feature. In NaOH, an oxidation feature (denoted *b* in Figure 9) with large hysteresis is followed by the onset of the OER. The oxidative feature likely corresponds to surface oxidation.

In summary, the inert potential region corresponds to potentials between −0.55 and 2.06 V vs. RHE, −1.03 and 2.20 V vs. RHE, and −0.72 and 1.94 V vs. RHE in 0.1 M H_2_SO_4_, 0.1 M NaAc, and 0.1 M NaOH, respectively. HOPG's excellent inert potential range in all three electrolytes makes it an ideal candidate substrate material for many studies. Like most of the other electrodes studied herein, the properties of the particular HOPG electrodes and the details of the experimental parameters used can influence the electrochemical behavior. Most notably, graphite step edge sites may have different reactivity than the basal plane sites [52], [80], so special care should be taken to assess the step edge density for applications where this parameter may be significant.

### Summary of Inert Potential Windows

**Figure 10:**
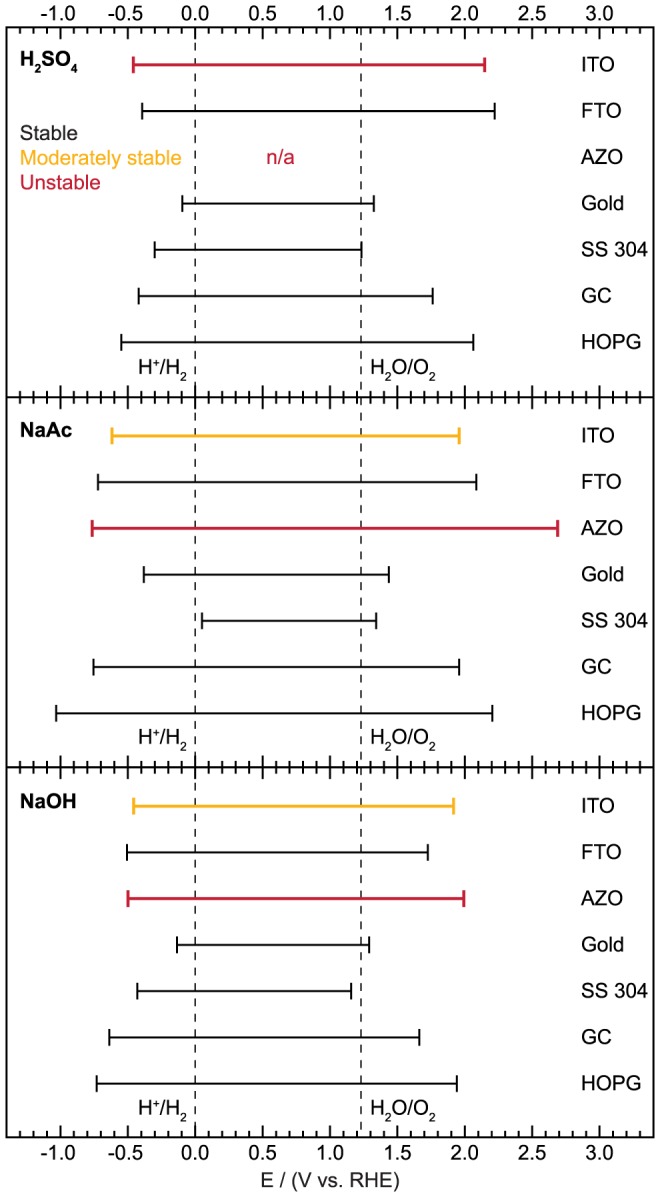
Potential range in which each substrate is inert for all electrolytes. Chemical stability is indicated by the color of the trace.

We employed a threshold current density of 50 µA/cm^2^ to determine the potential boundaries at which each substrate could no longer be considered electrochemically inert. Each substrate has a different value of capacitance so this 50 µA/cm^2^ was measured above the baseline capacitive current. Any initial transients were ignored. The actual potential at which this threshold is first reached was taken and the window of inertness of each substrate is shown in Figure 10. The chemical stability of the substrate in each electrolyte, relevant for longer term testing (>1 hr), is also indicated. In general, the TCOs have wide windows of inertness but are less stable than the opaque substrates. The GC and HOPG also draw very little current over a wide potential range and are very stable in all electrolytes. The results in Figure 10 should provide an excellent starting point for researchers in the selection of substrate materials for electrochemical studies. For example, FTO and ITO are suitable substrates for the study of thick, semiconducting photoelectrocatalysts while GC and HOPG are more appropriate for evaluating the activity of nanoparticulate or other low coverage catalysts. While some of these points were already known among experienced researchers in the field (though a substrate selection rationale is often omitted from published manuscripts), here we have quantified the useable potential windows for these important substrates to facilitate the substrate process for researchers in the future.

**Figure 11:**
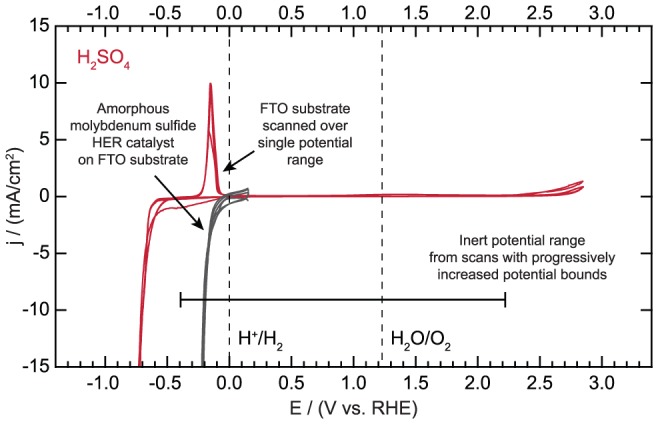
Fluorine-doped tin oxide (FTO) substrate scanned over a single potential range and hydrogen evolution catalyzed by amorphous molybdenum sulfide on FTO.

Our progressive scan methodology offers unique advantages over the more common technique of sweeping over a single, arbitrary potential range. The latter method can underestimate the window of inertness. Take for example the case of FTO in 0.1 M H_2_SO_4_; our results show that the substrate remains inert to a cathodic potential of −0.39 V vs. RHE. However, sweeping over a wider range without progressively increasing the potential bound could lead to a baseline scan such as is shown in Figure 11 where instead the cathodic bound appears to be 0.01 V vs. RHE. The large oxidative and reductive features could lead a researcher to erroneously conclude that this substrate is unsuitable to study HER catalysts whereas it is in fact appropriate for moderately to highly active catalysts. As shown in Figure 11, the activity of an amorphous molybdenum sulfide HER catalyst can be measured accurately when using FTO as the substrate [87]. This catalyst reaches a current density of 10 mA/cm^2^ at approximately −0.2 V vs. RHE. This value is in excellent agreement with a previous study which showed the same overpotential when the catalyst was deposited on glassy carbon [87]. A second key advantage to progressive scanning is the ability to associate oxidative features with corresponding reductive features as they develop. Take now the case of a gold substrate in NaOH. The progressive scanning method revealed that feature *c* denoted in Figure 6 was reduction of accumulated oxygen on the surface. If a single scan had been employed, it may not have been readily apparent that this feature resulted from oxygen reduction, and instead it may have been attributed to the reduction of gold oxide or another process. The substrate may have therefore been deemed unsuitable for use at any potentials positive of 0.35 V vs. RHE due to the presence of these large reductive features. However, using the progressive scanning methodology, we observed that this reductive feature arose only after the positive potential bound was increased sufficiently to drive oxygen evolution, which provided strong evidence that feature *c* resulted from oxygen reduction. Our results using the progressive scanning methodology show that gold is an acceptable substrate up to a potential of 1.29 V vs. RHE in 0.1 M NaOH. In short, progressive scanning of the substrate gives a researcher significantly more information to facilitate accurate analysis of the electrochemical data pertaining to the supported electrocatalyst or photoelectrode.

## Conclusions

The electrochemical activity and stability of several transparent conducting oxides (ITO, FTO, AZO) and opaque substrates (gold, SS, GC, HOPG) commonly used for evaluation of electrocatalysts and photoelectrodes have been evaluated. For each substrate, we identify the potential window in which the substrate is inert. While factors other than electrochemical inertness and stability, such as work function or surface termination, are also important to determine the appropriate substrate for a given application, the electrochemical properties of the substrate are almost always critical to consider for electrochemical applications. We therefore emphasize that each electrochemist should perform this type of baseline testing prior to electrocatalyst or photoelectrode evaluation. Due to the specific nature of the interactions between the substrate and electrolyte, some of the characteristic features may depend on the particular materials or experimental conditions employed. The results in this work provide a consistent basis for identifying viable substrates while the testing methodology reported herein provides a framework that can be used to make fair comparisons between potential substrates for their own studies.

## Supporting Information

File S1 **Area-normalized circuit resistance data as displayed in** **Figure 2** **.**
(XLSX)

File S2 **Electrochemical activity data for indium tin oxide (ITO) as displayed in** **Figure 3** **.**
(XLSX)

File S3 **Electrochemical activity and stability data for fluorine-doped tin oxide (FTO) as displayed in** **Figure 4** **.**
(XLSX)

File S4 **Electrochemical activity data for aluminum-doped zinc oxide (AZO) as displayed in** **Figure 5** **.**
(XLSX)

File S5 **Electrochemical activity data for gold as displayed in** **Figure 6** **.**
(XLSX)

File S6 **Electrochemical activity data for stainless steel 304 (SS 304) as displayed in** **Figure 7** **.**
(XLSX)

File S7 **Electrochemical activity data for glassy carbon (GC) as displayed in** **Figure 8** **.**
(XLSX)

File S8 **Electrochemical activity data for highly oriented pyrolytic graphite (HOPG) as displayed in** **Figure 9** **.**
(XLSX)

File S9 **Electrochemical activity data for fluorine-doped tin oxide (FTO) substrate scanned over a single potential range and hydrogen evolution catalyzed by amorphous molybdenum sulfide on FTO as displayed in** **Figure 11** **.**
(XLSX)
