# Correction: Substrate Selection for Fundamental Studies of Electrocatalysts and Photoelectrodes: Inert Potential Windows in Acidic, Neutral, and Basic Electrolyte

**DOI:** 10.1371/journal.pone.0127338

**Published:** 2015-04-30

**Authors:** Jesse D. Benck, Blaise A. Pinaud, Yelena Gorlin, Thomas F. Jaramillo

Fig 2 is incorrect. Please see the corrected Fig 2 here.

**Fig 2:**
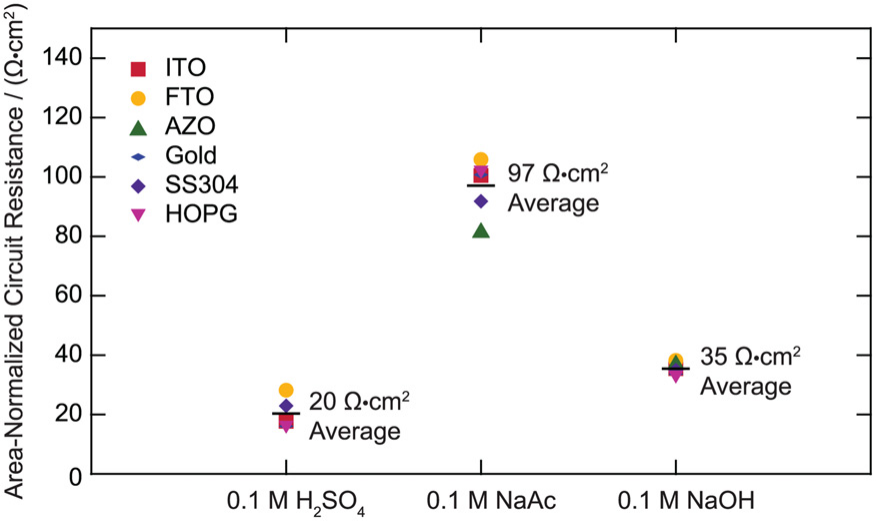
Area-normalized circuit resistances.

S1 File is incorrect. Please view the correct S1 File below.

## Supporting Information

S1 File Area-normalized circuit resistance data as displayed in Fig 2. (XLSX)
